# Dr. Charles Coote

**Published:** 1861-01

**Authors:** 


Dr. CHARLES COOTE was found dead in his bed on the thirteenth of November, death having apparently been occasioned by some cardiac affection. He was born in 1823, and having received his medical education in England, he afterward became a pupil of Virchow and Rokitansky. During the Crimean war, Dr. Coote was one of the Assistant Physicians to the British Civil Hospital at Smyrna, and at the time of his death was attached to the staff of the Middlesex Hospital.

